# Does an Apple a Day Also Keep the Microbes Away? The Interplay Between Diet, Microbiota, and Host Defense Peptides at the Intestinal Mucosal Barrier

**DOI:** 10.3389/fimmu.2020.01164

**Published:** 2020-06-09

**Authors:** Fabiola Puértolas-Balint, Bjoern O. Schroeder

**Affiliations:** ^1^Laboratory for Molecular Infection Medicine Sweden (MIMS) -The Nordic EMBL Partnership for Molecular Medicine, Umeå University, Umeå, Sweden; ^2^Department of Molecular Biology, Umeå University, Umeå, Sweden

**Keywords:** antimicrobial peptides, defensins, microbiota, diet, prebiotics and probiotics, high-fat diet, intestinal barrier function, gut bacteria

## Abstract

A crucial mechanism of intestinal defense includes the production and secretion of host defense peptides (HDPs). HDPs control pathogens and commensals at the intestinal interface by direct killing, by sequestering vital ions, or by causing bacterial cells to aggregate in the mucus layer. Accordingly, the combined activity of various HDPs neutralizes gut bacteria before reaching the mucosa and thus helps to maintain the homeostatic balance between the host and its microbes at the mucosal barrier. Defects in the mucosal barrier have been associated with various diseases that are on the rise in the Western world. These include metabolic diseases, such as obesity and type 2 diabetes, and inflammatory intestinal disorders, including ulcerative colitis and Crohn's disease, the two major entities of inflammatory bowel disease. While the etiology of these diseases is multifactorial, highly processed Western-style diet (WSD) that is rich in carbohydrates and fat and low in dietary fiber content, is considered to be a contributing lifestyle factor. As such, WSD does not only profoundly affect the resident microbes in the intestine, but can also directly alter HDP function, thereby potentially contributing to intestinal mucosal barrier dysfunction. In this review we aim to decipher the complex interaction between diet, microbiota, and HDPs. We discuss how HDP expression can be modulated by specific microbes and their metabolites as well as by dietary factors, including fibers, lipids, polyphenols and vitamins. We identify several dietary compounds that lead to reduced HDP function, but also factors that stimulate HDP production in the intestine. Furthermore, we argue that the effect of HDPs against commensal bacteria has been understudied when compared to pathogens, and that local environmental conditions also need to be considered. In addition, we discuss the known molecular mechanisms behind HDP modulation. We believe that a better understanding of the diet-microbiota-HDP interdependence will provide insights into factors underlying modern diseases and will help to identify potential dietary interventions or probiotic supplementation that can promote HDP-mediated intestinal barrier function in the Western gut.

## Introduction

The human gut is the interface between the body and the environment and is colonized by a community of trillions of microorganisms, including bacteria, fungi, and archaea. While the small intestine is responsible for nutrient absorption, the large intestine can rather be considered as a bioreactor in which gut bacteria carry out different biological functions, such as processing of dietary fibers (1, 2), maturation and regulation of the immune system (3, 4), and production of metabolites that exhibit various metabolic and neurological effects (5–7). At the intestinal interface, the immune system has the challenging task of maintaining a stable microbiota by keeping beneficial commensal bacteria at bay and by recognizing and eliminating disease-causing microbes. When this equilibrium is lost, the microbiota composition enters a state termed “dysbiosis,” which has been associated with a wide array of diseases.

Several environmental factors are known to directly alter or disturb the microbial composition, including diet and medicine use (8), but also intrinsic host factors such as host defense peptides (HDPs) (9), and host genetics (10). Therefore, strict regulation of immune signals in response to intrinsic and extrinsic stimuli is prompted at the intestinal interface to maintain homeostasis.

The intestinal defense system is composed of the gut associated lymphoid tissue (GALT), formed by a single layer of intestinal epithelial cells that are arranged in crypts and villi, and the underlying mesenteric lymph nodes and lamina propria. Goblet cells are dispersed over the epithelial layer and secrete mucus that functions as a physical barrier to maintain microorganisms at a safe distance from the intestinal epithelium. Immunoglobulin A (IgA)-secreting B-cells contribute to controlling local microbial communities (11). Paneth cells are specialized small intestinal cells at the bottom of the crypts of Lieberkühn that specialize in the production of HDPs, and together with enterocytes, which produce HDPs in the small and large intestine, they represent the primary source of HDPs in the gut.

HDPs are mostly small cationic peptides with unique mechanisms of action and different specificity against Gram-positive and Gram-negative bacteria (12). These antimicrobial molecules are the effector molecules of the intestinal immunity with potent bactericidal activity that has mostly been tested against intestinal pathogens (13). On the contrary, much less is known about how HDPs affect commensal bacteria, and several studies suggest that antimicrobial activity against the resident microbiota is comparably low or even absent (14–16). Yet, two independent studies demonstrated that transgenic intestinal expression or oral application of human alpha-defensin 5 (HD5) in mice could shape the intestinal microbiota composition *in vivo* (9, 17). It is therefore possible that previous activity testings of HDPs in *in vitro* assays did not appropriately reflect the *in vivo* conditions, as already demonstrated for human beta-defensins 1 (HBD1) and the Paneth cell-derived human alpha defensin 6 (HD6), which gained activity under adjusted conditions that reflected the intestinal microenvironment (18, 19). However, we are only about to begin to understand how HDPs affect commensal microbes and how the functionality of this defense system can influence the way the host copes with its inner microbial world in the intestine.

The secretion of Paneth cell HDPs can occur in response to bacterial stimuli and is largely regulated by signals from the transcription factor 4 (TCF-4)/Wnt signaling pathway in Paneth cells, while epithelial-derived HDPs rather seem to be controlled through IL-22, derived from immune cells (12, 20, 21). Microbial ligands are recognized through pattern recognition receptors (PPRs) present in intestinal epithelial cells or immune cells. Upon recognition and activation, immune cells of the GALT send signals to Paneth cells, goblet cells and enterocytes to coordinate their function and maintain the epithelial barrier function (22). In addition, the presence of microbes and their metabolites seems to be implicated in the control of the antimicrobial programming at the intestine, as germ-free (GF) mice have reduced HDP expression (23) and since probiotic supplementation or microbial-metabolite enrichment stimulates HDP production (24–27).

Members of the gut microbiota can influence HDP expression, and diet is considered one of the most influencing factors determining gut microbiota composition. Accordingly, diet composition, and whether it is of animal or plant-based origin, has profound implications in defining the gut microbial composition. For example, a diet rich in plant-derived fiber is associated with increased diversity in microbial communities, and more specifically, with an increase in *Bifidobacterium* abundance, which has been shown to be a positive regulator of intestinal barrier function (28, 29). As for proteins, animal-derived proteins were shown to decrease the abundance of Firmicutes, a phylum that has been associated with obesity and high body mass index (30), whereas plant-derived proteins were shown to promote the growth of beneficial *Bifidobacterium* and *Lactobacillus* genera and reduce the abundance of pathogenic bacteria (31). A Western-style diet (WSD), characterized by its low dietary fiber but high-fat and high carbohydrate content, markedly changes the microbiota composition in humans and mice (29, 32–35). Moreover, a WSD promotes a pro-inflammatory response through different dietary components (e.g., cholesterol, saturated and non-saturated fatty acids) and can cause microbiota-induced mucus defects as a result of the reduced fiber content (29, 36–38). Importantly, various studies indicate that the increased consumption of WSD in our modern societies, often accompanied by food additives such as artificial sweeteners and emulsifiers, is likely one of the drivers for the worldwide increase in non-communicable diseases, including metabolic syndrome and inflammatory bowel disease (IBD) (3, 39–42).

**Figure 1:**
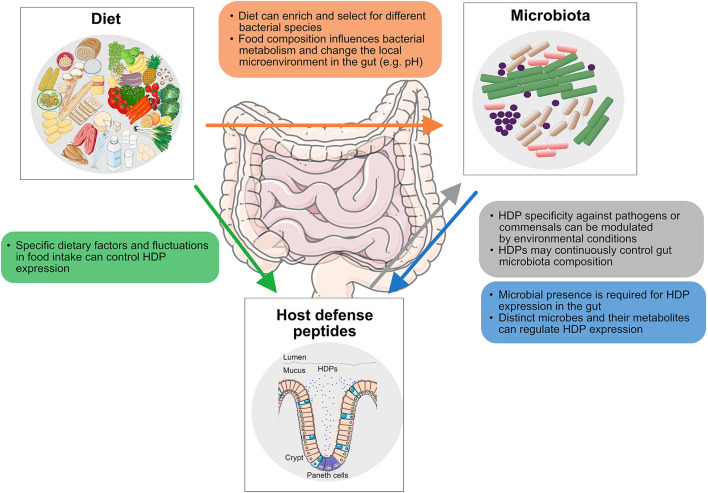
Interactions along the diet-HDP-microbiota axis discussed in this study. The impact of diet on gut microbiota composition (orange), the effect of diet on host defense peptide (HDP) expression (green), the activity of HDPs against gut microbes (gray), and the influence of bacteria and associated metabolites in HDP expression (blue) are displayed.

In this review, we summarize recent findings linking the effect of microbiota, diet, and food availability on the HDP-mediated intestinal defense function during intestinal homeostasis. Moreover, a defective HDP function has been linked to modern diseases associated with a Western-lifestyle, including IBD and metabolic disease. In particular, reduced levels of human defensins have been described in ileal Crohn's disease (43–45). Therefore, we also aim to decipher possible interactions along the diet-HDP-microbiota axis (Figure 1), that could be relevant in Western diseases, in which gut microbiota can gain access to the host epithelium due to an impaired barrier function. In that context, modulation of the gut microbiota through diet has been much discussed as a therapeutic alternative to protect the intestinal epithelium. Thus, we pose the question whether an apple a day keeps the microbes away, and we chose this fruit for several reasons: an apple is an easy-accessible every-day product that does not only contain fibers and polyphenols to potentially support the growth of HDP-stimulating bacteria, as we discuss below, but it was also recently shown that apples carry thousands of bacteria (46). Thus, it is theoretically possible that this fruit could serve as a natural pre- and pro-biotic to strengthen antimicrobial HDP function in the gut.

## Microbiota In Health and Disease

The human microbiota is generally dominated by the Bacteroidetes (*Bacteroides, Parabacteroides, Prevotella, Alistipes* genera) and Firmicutes (*Clostridium, Eubacterium, Blautia, Roseburia, Lactobacillus, Faecalibacterium, Ruminococcus, Streptococcus* genera) phyla. Other phyla, such as Proteobacteria *(Escherichia* genus), Actinobacteria (*Bifidobacterium* genus), and Verrucomicrobia (*Akkermansia* genus), are less represented and ratios of these phyla vary highly between individuals (47). A classification into “enterotype” groups was previously proposed, based on the function and relative abundance of the *Bacteroides, Prevotella*, and *Ruminococcus* genera within an individual, but the authors also stressed the fact that non-abundant species can exert high-abundant functions (e.g., methanogens), and that high-abundant microbes should thus not be regarded as solely responsible for the entire functionality of the human intestinal microbiota (48). Consequently, enterotypes do not seem to be as discrete as previously suggested, as they can be confounded by environmental variables, the clustering model used and stability over time (49).

Numerous studies have collectively attempted to define what constitutes a healthy microbiota, as the gut microbiota of healthy and diseased individuals differs in its composition. For example, the microbiota has been implicated in several disorders, such as IBD (50–52), obesity (53–56), diabetes (57–59), allergic diseases (60, 61), Parkinson's disease (62), autism spectrum disorder (63), and atherosclerosis (64), among others. Although there is in most cases no solid evidence that changes in the microbiota may cause these diseases, these microbial associations have encouraged the effort of finding strategies to modulate the microbial community composition through dietary intervention to improve the symptoms accompanying these disorders. Yet, the establishment of a healthy “ideal” microbiota is complex, as many factors are known to influence its composition (65). Here, we will focus on two key factors that are continuously affecting the intestinal microbiota, namely diet, and HDPs.

### Dietary Influence on Gut Microbiota Composition

**Figure 2:**
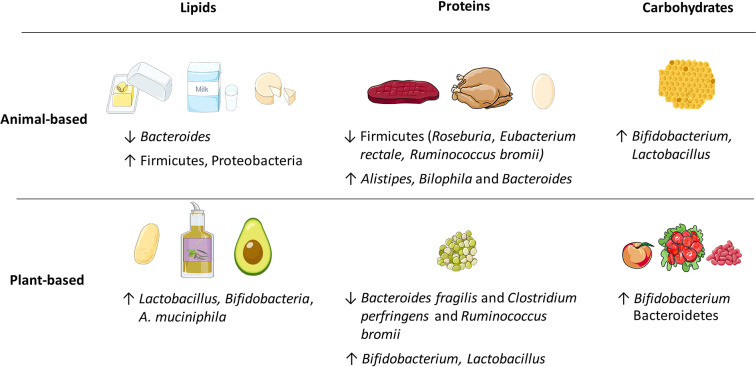
Examples of energy-delivering macronutrients, including lipids, proteins, and carbohydrates that produce various changes in the relative abundance of gut microbiota. The animal or plant-based origin dramatically influences the outcomes.

The impact of different diets on the intestinal microbiota has been extensively reviewed in recent years (28, 30, 31, 66). The composition of the diet (defined by macronutrient ratio—carbohydrates, fats and proteins), the origin of these components (plant or animal-based) and the availability of different dietary factors are recognized as determinants of gut microbial metabolism and composition, with the potential to influence human health (67, 68) (Figure 2). The three major macronutrients carbohydrates, fats and proteins, can reach the colon after escaping the primary digestion in the small intestine when the intake surpasses the rate of digestion, or due to the biomolecules' intrinsic structural complexity (69–71). Therefore, the proportion of macronutrients present in, for example, a Western-style, protein-rich, vegan, vegetarian, or fiber-rich diet will have different effects on the colonic microbiota.

#### Fiber

The dietary compound that has been found to be the strongest contributor to gut microbial community structure is dietary fiber. Dietary fiber is almost exclusively of plant-based origin and can be found as soluble or insoluble carbohydrate polymers that are inaccessible to the human body due to the limited number (ca. 17) of carbohydrate-active enzymes (CAZymes) (2). In contrast, it is estimated that the gut microbiota is equipped with 11,000 CAZymes that carry out the hydrolysis of different sets of soluble fibers (72, 73), also known as microbiota-accessible carbohydrates (MACs) (2). As a result of bacterial fiber fermentation, short chain fatty acids (SCFAs), including acetate, propionate, and butyrate, and gases such as H_2_ and CO_2_, are produced by different gut bacteria in a complex cross-feeding network. Enterocytes utilize SCFAs as an energy substrate, and these metabolites have also been shown to improve the intestinal barrier integrity, regulate glucose homeostasis and lipid metabolism, and induce both anti-inflammatory and tolerogenic immune reactions (74). Conversely, insoluble fibers are not fermented by the microbiota and do not convey the aforementioned benefits.

Microbial fermentation is determined by the origin, chemical composition and physicochemical properties of the fibers present in food (28). As a rare example of animal-derived carbohydrate, honey includes a diverse mixture of mono- and disaccharides as well as complex carbohydrates. Although the effect will depend on the specific type, honey was shown to promote the growth of *Bifidobacterium* and *Lactobacillus* (75). Fibers that originate from plants—derived from either cereals, grains, vegetables, legumes or nuts—have unique chemical compositions and physicochemical properties (28). Therefore, the variety of fibers present in plant-based diets can support more diverse gut microbial communities (76, 77).

Fruits are another common source of plant-derived fibers. For example, complex pectins found in apples and wine can be degraded by *Bacteroides thetaiotaomicron* (*B. thetaiotaomicron*) (78), and kiwifruit supplements increased the abundance of *Faecalibacterium prausnitzii* (*F. prausnitzii*) in patients with constipation (79), while a crude extract of kiwi was shown to support the growth of *Bifidobacterium* and *Bacteroides in vitro* (80).

Clinical studies assessing the impact of different types of fibers on the microbiota report that *Bifidobacterium spp*. are enriched following consumption of diets with certain fibers, including galacto-oligosaccharides (GOS), inulin-type fructans, xylo-oligosaccharides, and arabinoxylan-oligosaccharides, and that microbes in the Bacteroidetes and Firmicutes phyla are differentially stimulated by soluble fibers from corn or polydextrose (28, 81). In addition, studies comparing the low-fiber diet of Westernized populations with the high-fiber diet of unindustrialized communities show dramatic differences in the microbiota composition between both populations (34, 35), including that the Westernized societies having decreased diversity and apparent loss of certain microbes that are present in the unindustrialized communities (82).

#### Lipids

A high-fat WSD, mainly containing saturated or trans-fat, is associated with a decrease in *Bacteroides* and an increase in Firmicutes and Proteobacteria relative abundance (34, 83–85). Conversely, mono- and polyunsaturated fat present in low levels in vegan/vegetarian diets increase the levels of lactic acid bacteria, *Bifidobacteria*, and *Akkermansia muciniphila* (*A. muciniphila*) (30, 31). In mice, both lard-based and palm-oil based HFDs increased the relative abundance of the *Clostridiales* and *Bacteroidales* classes in specific pathogen free (SPF) mice (86). However, no significant differences in microbiota composition were observed between both diets that mainly differ in their cholesterol content, where a lard-based diet contains 10 times more cholesterol than the palm-oil based HFD (86).

Agans et al. demonstrated in an *in vitro* multi-vessel analysis that distinct gut microbiota can utilize dietary fatty acids as a sole carbon source through β-oxidation and anaerobic respiration pathways (87). Thus, bacteria that possess fatty acid oxidation enzymes, for example *Alistipe*s *spp*. and members of the Proteobacteria phylum (*Bilophila, Escherichia/Shigella, Citrobacter*, and *Enterobacter spp*.) were enriched in a medium containing only capric acid, palmitic acid, stearic acid, oleic acid, and linoleic acid (87). Interestingly, however, in the small intestine, where most of the macronutrient digestion and absorption occurs, the intestinal microbiota was also shown to be capable of regulating host dietary fat digestion and absorption in mice (88). In that study, consumption of a HFD increased the relative abundance of the *Clostridiaceae* family at the mucosa, most markedly in the jejunum and ileum, and one member of this family was shown to secrete an unknown metabolite capable of mediating lipid absorption (88). Thus, the mucosa-associated microbiota can be highly sensitive to dietary lipid changes and can play an important role in nutrient absorption.

#### Proteins

In addition to fiber fermentation, protein metabolism by bacteria can produce a small fraction of SCFAs too, but also more detrimental metabolites originating from animal diets (eggs, beef, pork). For example, the food-derived microbial metabolite trimethylamine N-oxide (TMAO) is linked to cardiovascular disease and atherosclerosis (89, 90). Moreover, a diet rich in animal protein is associated with a decrease in members of Firmicutes phylum that are known to metabolize plant polysaccharides (e.g., *Roseburia, Eubacterium rectale*, and *Ruminococcus bromii*) and with an increase in the levels of bile-tolerant bacteria (*Alistipes, Bilophila*, and *Bacteroides*) (67, 91). However, individuals consuming pea protein—a plant-based alternative for meat—displayed increased intestinal SCFA levels and a bloom in beneficial *Bifidobacterium* and *Lactobacillus*, while pathogenic *Bacteroides fragilis* and *Clostridium perfringens* levels were reduced (31). Furthermore, observations in protein supplementation studies showed an increase in the total amount of bacteria, as determined by absolute-abundance (92). This was proposed to be linked to the increased availability of nitrogen, an otherwise limited nutrient in the gut, as a result of the higher protein intake (92).

#### Other: Micronutrients and Food Additives

Besides the discussed macronutrients, micronutrients are increasingly acknowledged to influence the gut microbiota. Some of these compounds transit the small intestine, where a large number of digestible nutrients are already absorbed, and reach the colon intact, where they concentrate and interact with the microbiota (66, 93). Examples of micronutrients include polyphenols—naturally occurring plant metabolites—(e.g., lignans, isoflavones, stilbenes), trace elements and vitamins. Polyphenols in plant-based diets are generally considered to have a prebiotic effect, i.e., supporting the growth of beneficial bacteria such as *Bifidobacterium* and *Lactobacillus* (94–96), can be antimicrobial against different bacterial pathogens and can have anti-inflammatory effects (31, 97). Trace-elements, such as iron and zinc, have a low abundance in the gut and are thus competed for amongst pathogens and commensals, thereby also affecting gut microbial composition and/or favoring pathogen colonization (98–100). Other micronutrients such as vitamins B6 and B12 serve as cofactors for microbial enzymes and consequently, gut microbial species compete with the host for these diet-derived vitamins in the small intestine (101).

Remarkably, food additives that are often present in modern diets (e.g., non-caloric artificial sweeteners (NAS) such as sucralose, saccharin and aspartame) and emulsifiers [e.g., carboxymethyl cellulose (CMC) and polysorbate-80 (P80)], induce significant dysbiosis. The microbiota of NAS-consuming mice provoked an overgrowth of *Bacteroides spp*. and reduced levels of *A. muciniphila* (39). Emulsifier-treated mice had a reduction in the *Bacteroidales* population and an increase of the mucolytic bacterium *Ruminococcus gnavus*, which was accompanied by decreased SCFA production and the development of metabolic syndrome (40).

In summary, individual macronutrients and micronutrients can have distinct effects on gut microbiota composition, which in turn can have subsequent effects on human health. However, caution is prompted when linking a phylum to a specific diet, given the dynamic nature of the microbiome and due to the challenging task of disentangling which dietary effector in the complex composition of the diet is driving the observed changes (66). For example, the changes in gut microbial composition observed in HFDs could be biased by the low dietary fiber content and may not be a direct consequence of the fat content or composition. Indeed, Morrison et al. showed that switching from a regular chow diet to a refined low-fat/low soluble fiber diet was accountable for the change in the fecal community structure in mice (102). In contrast, a switch from the low-fat/low soluble fiber diet to a low soluble fiber/HFD kept the initial observed changes without further alteration. Remarkably, the authors observed expansion of *Clostridia* and Proteobacteria and a reduction of Bacteroidetes when switching from a chow diet to a diet low in fat and lacking soluble fibers; these alterations are typical of HFD interventions (102).

## Intestinal HDPs as Key Effectors of Mucosal Barrier Function

**Figure 3:**
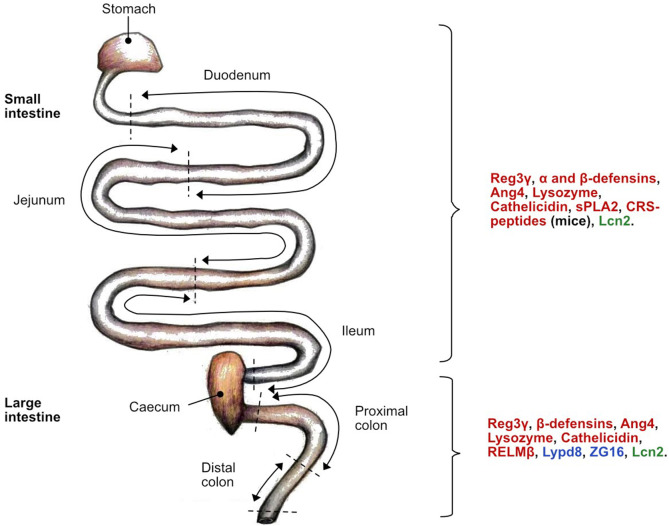
Location of host defense peptide (HDP) expression along the mice intestine under normal physiological conditions. The antimicrobial activity is specific to each HDP class: bactericidal (red), bacteriostatic (green) and aggregation (blue). α-defensins can also be referred to as Defas in mice. C-type lectin regenerating islet-derived protein 3 gamma (Reg3γ); Angiogenin 4 (Ang4); secretory phospholipase A2 group IIA (sPLA2); cryptdin-related sequence peptides (CRS-peptide)s; Lipocalin-2 (Lcn2); Ly6/PLAUR domain containing 8 protein (Lypd8); zymogen granule protein 16 (ZG16); resistin-like molecule beta (RELMβ).

Besides the nutrients derived from the ingested food, the host also plays an active role in shaping the gut microbial community. While the production of IgA and mucus as modulators of gut microbiota composition have been discussed elsewhere (103, 104), we will here focus on the release of HDPs, which due to their positive charge are retained in the intestinal mucus layer (105–107). Intestinal HDPs are a diverse group of proteins that possess unique mechanisms of action and spectrum of activity against microbes. In part, these mechanisms depend on the HDP localization in the intestine (Figure 3) and the specific regulatory mechanisms of expression and activation (12).

### Location

In the intestine, different epithelial cell subsets produce distinctive HDPs. Enterocytes produce the regenerating islet-derived protein 3-gamma (Reg3γ) and β-defensins in both the small and large intestine, and the Ly6/PLAUR domain containing 8 protein (Lypd8) exclusively in the large intestine (108). While the primary function of goblet cells relies on the production and secretion of MUC2, they also secrete resistin-like molecule beta (RELMβ), an HDP that is active predominantly in the colon (109, 110). However, the vast majority of HDPs are produced by small-intestinal Paneth cells and include lysozyme, α- and β-defensins (α-defensins are alternatively called cryptdins in mice), angiogenin-4 (Ang4), secretory phospholipase A2 group IIA (sPLA2), and Reg3γ. Although mature Paneth cell HDPs have been isolated from the large intestine, they probably also originate from small-intestinal Paneth cells (111).

Immune cells also contribute to the HDP repertoire with secretion of lipocalin-2 (Lcn2) by neutrophils and lysozyme by macrophages (112). Also, neutrophils produce human neutrophil peptides (HNPs), a class of α-defensins, which are only produced by humans and not by mice (113). To compensate for this, however, mice seemingly evolved and acquired an additional set of peptides closely related to α-defensins, called cryptdin-related sequence (CRS) peptides, that are also produced by Paneth cells (114).

### Antimicrobial Activity of HDPs

Defensins possess a broad spectrum of antimicrobial activity, as *in vitro* studies demonstrated that they are bactericidal against Gram-positive and Gram-negative bacteria, fungi, viruses, and unicellular parasites (115). These small secreted peptides (30–40 amino acids long, 3 to 5 kDa) are characterized by six conserved cysteine residues that form three disulfide bridges (116). The in-sequence linkage of the cysteines distinguishes α- and β-defensins, the two largest defensin families (113). The cationic nature of these peptides advantageously attracts them to the negatively charged outer envelope of bacteria, produced by the presence of phospholipids in Gram-negatives and of teichoic acid in Gram-positives. In its majority, defensins are thought to act by disrupting the bacterial membrane integrity or via inhibition of the cell wall synthesis by interacting with lipid II (112, 113, 117). Interestingly, due to this cationic property, HDPs have recently been attributed different tumor killing capabilities, as the membrane of tumor cells have increased expression of negatively charged cell surface glycoproteins (118).

Mice possess a wide array of α-defensin and CRS-peptides in the intestine, both with many gene paralogs and high sequence similarity between different laboratory mouse strains, which complicates research on these molecules (119, 120). In addition, expression levels of mice defensins vary along the small intestine (23). α-defensins, also called cryptdins in mice, exhibit a variable spectrum of activity depending on their oxidation status (discussed below); for example displayed reduced α-defensin 4 a greater antimicrobial activity against 7 different commensal bacteria than the oxidized form (15). Furthermore, the antimicrobial activity spectrum of CRS-peptides relies on their characteristic ability to form covalent disulfide-bridged homo- and heterodimers, conferring different killing capabilities against commensal *Enterococcus faecalis (E. faecalis)* and *Lactobacillus fermentum* and the pathogens *Streptococcus pyogenes, Listeria monocytogenes, Escherichia coli* (*E. coli*) and *Salmonella typhimuriu*m (*S. typhimurium*) (114).

In humans, the most abundant intestinal HDPs are HD5 and HD6 (121). The potent antimicrobial activity of HD5 was shown against *Staphylococcus aureus* (*S. aureus*) and *E. coli*, which was comparable to the activity of HNP2 and HNP4, respectively (122). HD6 was shown to self-assemble and acquire a nanonet formation, capable of entrapping bacteria (123), and of disrupting the cell envelope of bacteria in a reducing environment (19). Other intestinal HDPs in humans include the β-defensins HBD1, HBD2, and HBD3. Interestingly, HBD1 seems to be constitutively expressed whereas HBD2 and HBD3 can be induced by microbial products (116, 124). HBD1 and HBD3 were found in human rectal-mucus extracts and their activity was not affected by binding to mucus (107). HBD3 possesses a broad spectrum of activity against facultative anaerobic commensal and pathogenic bacteria (19). Of note, HBD1 can exist as an oxidized, disulfide-bridged form and a reduced, linear peptide, which differ in their antimicrobial activity spectrum: reduced HBD1 exhibits a higher antimicrobial effect against the opportunistic pathogen *Candida albicans* (*C. albicans*) and different commensal *Bifidobacterium* and *Lactobacillus* strains when compared to its oxidized form (18). Recently, it was shown that even an HBD1-derived octapeptide fragment that was generated through digestion by gastrointestinal proteases caused cell wall and membrane defects as well as the disintegration of cytosolic structures of *E. coli* and *C. albicans* (125).

Further HDP classes include Reg proteins, angiogenins, and Lcn2. Reg3γ in mice (or the human homolog Reg3A, previously known as human HIP/PAP), present antimicrobial activity against Gram-positive bacteria via formation of an hexameric pore structure in the bacterial membranes (126). Interestingly, angiogenins have been attributed to different functions, including tumorigenesis, cell growth, and apoptosis, and Ang4 was shown to be bactericidal against Gram-positive *E. faecalis* and *L. monocytogenes* through a yet unknown mechanism of action (127, 128). Lcn2 is mainly secreted by neutrophils and prevents bacterial growth by sequestering iron-scavenging siderophores (129). Additionally, in the absence of Lcn2, mice were more susceptible to bacterial colonization, not only owing to the reduction of the bactericidal effect but also by altering the migration of neutrophils and reducing the expression of cytokines by macrophages (130).

ZG16, Lypd8, and RELMβ have been described as peptides that maintain the spatial segregation between the gut microbiota and the hosts' epithelial cell surface in the colonic mucus layer (108, 110, 131). ZG16 is a highly abundant protein in the colon that intertwines with the mucin polymeric network and contributes to space separation by binding to Gram-positive bacteria (131). Lypd8, a highly glycosylated glycosylphosphatidylinositol-anchored protein, binds flagellated bacteria and was recently shown to inhibit the attachment of *Citrobacter rodentium* to epithelial cells through competitive binding of bacterial intimin, thereby interrupting its interaction with the translocated intimin receptor (Tir) in the host (108, 132). Lypd8 thus specializes in inhibiting the colonization of intestinal pathogens. RELMβ controls the levels of Gram-negative Proteobacteria in the inner colonic mucus layer of mice by forming pores into the bacterial cell membrane (110). Of note, the human homolog (hRETN) was shown to be specifically bactericidal against Gram-negative pathogens but lacked activity against commensal bacteria such as *E. faecalis* and *B. thetaiotaomicron* (110). Additionally, RELMβ is inducible by the microbiota and was shown to be elevated during intestinal inflammation and potentially also aging, as observed in 104 weeks old mice (133, 134).

Altogether, there is reason to believe that HDPs in the small intestine are mainly aiming to “kill” bacteria, whereas those in the large intestine have a more spatial segregation function and are not necessarily bactericidal.

### Transcriptional Regulation of HDPs

Key to the antimicrobial effect of HDPs are the regulatory cues behind their function, which can occur at the level of granule-release, expression, and activation. For example, defensin exocytosis from Paneth cell granules is mediated by the ATG16L1 and ATG5 proteins of the autophagy pathway (135), and can also occur in response to bacterial stimuli (21), which will be discussed later. In the crypts of the small intestine, stem cells differentiate into Paneth cells via the Wnt/β-catenin signaling pathway under the activity of the transcription factor TCF-4 (20, 136). Studies performed in mice show that the Paneth cell maturation process is accompanied by the appearance of α-defensins and CRS-peptides during the weaning period (23, 137). Moreover, TCF-4 can control the expression of α-defensins and CRS-peptides in mice and humans—as the promotor region of these genes has binding sites for TCF-4, suggesting their baseline release levels occur in parallel to Paneth cell differentiation (20, 119). Recently, the pro-inflammatory cytokine interferon gamma (IFN-γ) was also identified as a potent inducer of Paneth cell degranulation and goblet cell mucus production in a model of murine primary organoid culture (138).

Although several studies report that Paneth cells and intestinal epithelial cells can directly respond to bacterial stimuli, immune cell-derived signals are the main effectors triggering HDP expression (138). Upon recognition of microbe-associated molecular patterns (MAMPs) by Toll-like receptors (TLRs) present in innate lymphoid cells (ILCs) and dendritic cells, downstream signaling orchestrates an inflammatory response and integrates different signals oriented to control the expression of different HDPs. Activation of the NLRP6 inflammasome signaling by the microbiota—as shown in GF vs. SPF mice—directed the release of IL-18, which induced the HDPs intelectin 1a (ITLN1), RELMβ, and Ang4, implicating IL-18 as a regulator of the antimicrobial program of the colonic mucosa (27). Likewise, IL-25, a Th2 cytokine mainly known for its anti-helminth function, was shown to also induce the expression of *Ang4* in an IL-23 dependent manner (139). Furthermore, IL-22 has been demonstrated to induce *Reg3*β, *Reg3*γ, β-defensins, *Lcn2*, and *Ang4* (12, 12, 140–144), as well as mucin production (145). The cellular sources of IL-22 include type 3 innate lymphoid (ILC3), natural killer (NK), Th17 and Th22 cells as well as dendritic cells (146–148).

Whereas, Reg3γ is induced by the microbiota-dependent inflammatory signals of the TLR-MyD88-IL-22 axis (24, 120, 143), evidence for a role of MyD88 in α-defensin regulation is conflicting. Castillo *et al*. reported that MyD88 is not involved in defensin regulation, as the total defensin copy number in the small intestine of *Myd88*^−/−^ mice, was not different from the *Myd88*+/+ control group (120). Conversely, Menendez *et al*. showed a drop in ileal defensin (*Defa*) expression in *MyD88*^−/−^ mice, using a relative expression approach (149). Likewise, Liang *et al*. showed that *MyD88*^−/−^ mice had reduced gene expression of *IL-22*, and of *Mmp7*, the coding gene for the matrix metalloproteinase-7 (Mmp7) which is a key enzyme that is required to activate mouse α-defensins (discussed below), and diminished mature α-defensins under normal conditions (150). These last observations suggest that MyD88 regulates *Mmp7* expression, and therefore the post-transcriptional activation of α-defensins (150). Thus, MyD88 may not regulate transcription of *Defa* genes directly, but affect α-defensins rather indirectly through regulation of Mmp7. However, this explanation does not provide reasoning to the transcriptional downregulation of *Defa* observed by Menendez *et al*. (149). Finally, most recently the TIR domain-containing adaptor molecule 1 (TRIF or TICAM1) was described as a key homeostatic regulator of epithelial barrier function by controlling the expression and protein levels of Mmp7, Reg3γ, and Defa1 (151). In this study, *MyD88*^−/−^ mice again had no influence on *Defa1* expression and showed only a slight reduction in *Mmp7* expression (151).

### Post-transcriptional Regulation of HDPs

On the activation level, environmental conditions and the presence of different proteases can determine the activity of defensins. HDPs with membrane-lysing capacities can be toxic to eukaryotic cells. Because of this, defensins are secreted as pro-peptides that are processed and activated in the gut lumen by Mmp7, also known as matrilysin, in mice and by trypsin in humans (152, 153). Cutting off the pro-region will activate their antimicrobial function, and once in its mature form, some peptides can resist proteolysis, as was shown for α-defensin 4 (111). In the gut, HDPs can be further processed by other proteases such as gelatinase (*GelE*) or serine protease E (*SprE*), secreted by *E. faecalis*, or chymotrypsin and neutrophil elastase, produced by the host (111).

Another form of regulation of HDP activity relies on the local microenvironment in the intestine. Defensins form disulfide bridges that can modulate the antimicrobial effect, depending on whether they are in their reduced (linear/open) or oxidized (closed) forms, as exemplified for α-defensin 4 (15). Redox-potential and pH differ between the bottom of the intestinal crypts (where most HDPs are secreted) and the gut lumen and can thus shape the tertiary structure of α-defensins. Furthermore, the enzymatic thioredoxin system in the gut is a host-dependent mechanism to control redox reactions, and this system mediated the reduction of HBD1, thereby revealing its potent antimicrobial activity against common anaerobic gut bacteria (18). Of note, antimicrobial activity of α- and β-defensins was differently modulated when conditions were adjusted for pH and redox-potential, and was independent of bacterial genus, cell wall composition, or defensin class (19). In this manner, a reducing environment exposed a bactericidal effect of HD6, while the nanonet conformation was maintained under these conditions (19). Importantly, in the gut the reduced or oxidized conformations will be subject to protease activity to either activate or deactivate them (125, 154). While oxidized HBD1 and HD5 were resistant to protein digestion, HBD1_red_ was readily digested *in vitro* and generated a C-terminal octapeptide (125). This octapeptide gradually lost its activity in acidic conditions, further highlighting the influence of environmental regulation on HDP activity (125). Similarly, HD5_red_ was efficiently degraded by host proteases and produced ten new fragments, some of which exhibited an antimicrobial effect against commensal bacteria, and thus greatly increased the known spectrum of activity of this peptide (154). In contrast, HD6_red_ was unaffected by host proteases, mainly due to its characteristic nanonet formation (154).

In summary, the antimicrobial activity of intestinal HDPs is controlled by a complex interplay between transcriptional and post-transcriptional signals that are central to maintaining homeostasis in the gut (12). Some of these regulatory factors are under the influence of the host, but other factors tightly depend on the presence and the composition of the gut microbiota. While HDPs have historically been considered to protect the host against pathogens, many studies preferentially included pathogenic bacteria and fungi in their antibiotic activity tests. It is thus possible that the activity against anaerobic, commensal bacteria is underestimated, due to a study bias and due to the selection of simple testing conditions that did not resemble the conditions in the gut. In fact, only by modulating some of the environmental parameters in the activity tests, the antimicrobial effect of several HDPs against commensal bacteria could be revealed (15, 18, 19, 154). It is thus required to keep these factors in mind in order to increase our understanding of the function of these peptides in shaping microbial communities in the gut.

## Impact of HDPs on Intestinal Microbiota

Intestinal HDPs protect the host against microbial intruders in the gut and have the potential to shape the intestinal microbiota (155) (Table 1). Specifically, the HDP family of defensins has been shown to exert noticeable effects on gut microbiota composition. Transgenic mice expressing HD5 on top of their indigenous HDP repertoire were shown to have an expansion of the Bacteroidetes and a reduction of the Firmicutes phyla in the small intestine (9). Interestingly, a fragment produced after proteolysis of HD5 (HD5_1−9_), shifted the fecal microbiota composition and influenced the microbial diversity in the small intestine, specifically increasing *Akkermansia* and a member of the Ruminococcaceae family and decreasing *Intestimonas* from the Clostridiaceae family (154). Furthermore, administration of HD5 in a murine model of diet-induced obesity reversed dyslipidemia and improved the overall glucose regulation (17). The latter was partly attributed to HD5-induced changes in the fecal microbiota, namely an increase in *Bifidobacterium* and *Alloprevotella* abundance, that correlated with improved metabolic parameters (17). However, the effect on microbiota composition in the small intestine was less marked. Thus, both the transgenic expression and administration of defensins can shape the microbial communities in the mouse intestine.

**Table 1:** Influence of host defense peptides (HDP) on microbiota composition in *in vivo* studies.

**Host defense peptide treatment**	**Effect on SI microbiota (relative abundance)**	**Effect on fecal/colonic microbiota (relative abundance)**	**References**
Transgenic expression of HD5	↑ Bacteroidetes ↓ Firmicutes	NA	(9)
Mmp7^−/−^	↑ Firmicutes ↓ Bacteroidetes	NA	(9)
	NA	No difference when compared to wild-type	(111)
HD5_1−9_	↑ *Akkermansia* and members of the Ruminococcaceae family ↓ *Intestimonas* from the Clostridiaceae family	↓ *Bacteroides* and *Lactobacillus* genera ↑ *Akkermansia spp*. and *Parasutterella*	(154)
Administration of HD5	↑ *Bifidobacterium*	↑ *Bifidobacterium* and *Alloprevotella* ↓ *Parabacteroides*	(17)

*NA, not available, was not investigated in the study. SI, small intestine*.

The impact of mice defensins on gut microbial communities was also studied in mice lacking Mmp7 (152). *Mmp7*^−/−^ mice have an increase in the levels of Firmicutes (mainly *Clostridia*) and a significantly lower proportion and abundance of *Bacteroides* in the small intestinal microbiota, which is contrary to the result observed in mice expressing HD5 (9). As both the HD5 transgenic and the *Mmp7*^−/−^ mice models showed no effect on the total bacterial numbers, the defensin function seems likely restricted to shaping the composition of the microbiota rather than controlling its abundance in the small intestinal lumen. Interestingly, and in contrast to the small intestine, the caecal and colonic microbiota of *Mmp7*^−/−^ mice were not different from their wild-type controls (111). These findings were attributed to the existence of other host and microbial proteases present in the large intestine that could convert inactive defensin precursors into active peptides, thereby explaining the lack of an effect on the microbiota composition at this intestinal site (111).

However, as discussed above, the overall impact of HDPs on gut microbiota community structure is expected to be reflected by the potential antimicrobial effect against gut commensals. And indeed, administration of HD5 increased *Bifidobacterium* relative abundance (17), while transgenic expression of HD5 on top of the own mouse antimicrobial arsenal led to increased relative abundance of Bacteroidetes (9). This suggests that defensins can promote the growth of selected microbial taxa by specifically eliminating other microbes. While some HDPs, such as hRETN, human LL37 or mouse cathelicidin related antimicrobial peptide (CRAMP) show no antimicrobial effect against commensal microbes (16, 110), defensins have anti-commensal activity that was dependent on an environment that resembled the conditions in the gut (15, 18, 19). Regardless of this, most *in vitro* studies have investigated the antimicrobial effect against isolated pathogens and not against complex communities of commensals. Accordingly, these findings have contributed to build up the notion that HDPs do not affect commensals. Therefore, more work is required to determine the effect of several HDP classes with adjusted microenvironmental conditions and to truly understand the impact of HDPs on the gut microbiota community.

### Altered HDP Expression in Microbiota-Associated Diseases

An indication that HDP modulation of gut microbiota composition might also be true in humans is based on the fact that gut-associated inflammatory disorders are commonly accompanied by dysbiotic communities, and that ileal Crohn's disease, a form of IBD, can be linked to Paneth cell dysfunction (156–158). Accordingly, in two German cohorts patients with ileal Crohn's disease had reduced levels of HD5 and HD6, which was even more pronounced in patients carrying a mutation in the intracellular nucleotide binding oligomerization domain 2 (NOD2) receptor (43, 44). However, in another cohort in Australia, reduced levels of HD5 were associated with inflammation and not with the NOD2 genetic status (45). Yet, despite that Paneth cells are the predominant cell types expressing NOD2 at the intestinal mucosa, it is unclear whether mutations in NOD2 are indeed causing the altered expression of the human defensins in ileal Crohn's disease (159).

In addition to NOD2, other genetic risk factors that may affect Paneth cell function in Crohn's disease include mutations in ATG16L1 (160), which is part of the autophagy pathway and implicated in Paneth cell degranulation, or in the transcription factor X-box binding protein-1 (Xbp1), a key regulator of the endoplasmic reticulum (ER) stress response (161). Mutations in Xbp1 can result in ER stress, defects in Paneth cell granule morphology and reduced lysozyme levels, leading to intestinal inflammation (162).

Besides patients with ileal Crohn's disease, patients with obesity (BMI>35) evidenced defective Paneth cell secretions, which was linked to the activation of the unfolded protein response (UPR) during ER stress (163). Even though the morphology and number of Paneth cells were normal, these individuals had reduced HD5 and lysozyme protein levels, despite having an increased gene expression of these HDPs, suggesting the presence of transcriptional arrest (163). Similarly in mice, an obesogenic diet was associated with Paneth- and goblet cell abnormalities, a worsened colonic inflammation and expansion of *Atopobium spp*. and Proteobacteria in the fecal microbiota (164). Taken together, a defective antimicrobial defense that cannot sufficiently control microbial communities in the gut may likely be a contributing factor in the pathogenesis of ileal Crohn's disease and obesity. However, it is still not fully clear if defects in defensin expression or secretion precede or follow the onset of disease.

## Direct Effect of Diet on HDP Expression

We have described the implications of different dietary compounds on the microbial communities in the gut. To add to this function, evidence of a direct effect of diet (i.e., the presence of certain dietary components or the impact of complex diets) and nutritional status (i.e., fasting or starvation condition) on HDP function, has been accumulating over the past decade. Takakuwa *et al*. tested 20 amino acids in mice-derived enteroids to investigate their defensin-inducing capacity *in vitro*. The release of Defa1 was strongly induced by leucine, and to a lesser extent, by tryptophan (165). Their observations thus suggest a direct role of distinct amino acids in the induction of defensin expression by intestinal cells. Another study explored how diets supplemented with kidney bean flour, which is rich in fiber and phenolic compounds, influenced colonic barrier function in a mouse model of colitis. When compared with a basal control diet, the dietary flour intervention increased SCFAs (acetate, butyrate, and propionate) and up-regulated MUC1 and RELMβ in unchallenged mice (166). This effect was more pronounced after the induction of colitis and at the same time, the bean flour treatment decreased the expression of pro-inflammatory cytokines and improved colitis symptoms (166). Moreover, Bentley-Hewitt *et al*. demonstrated that *in vitro* fermented kiwifruit products significantly increased the production of HBD1 and HBD2 by intestinal epithelial cells. This effect, which was mainly mediated by the production of SCFAs and was not observed after treatment with the digested kiwifruit lacking fermentation products, suggests that the HDP modulating effect was exerted by fermentation products and not directly by the digested kiwifruit (167).

### Food Availability

In addition to individual components of the diet (i.e., food quality), food availability (i.e., food quantity) has also been linked to HDP function. Mice deprived of food for 48 h had reduced expression of the Paneth cell antimicrobial peptides lysozyme, defensins, and *Reg3*γ, which was confirmed on the protein level for the precursor of Reg3γ and lysozyme (168). Although the numbers of Paneth cells were not changed, the physiology of their granules was altered. Furthermore, a 2-fold increase in bacterial translocation to the mesenteric lymph nodes was observed in the starved mice (168). Interestingly, the same authors showed opposite results in a second study, in which they evaluated the impact of total parenteral nutrition on Paneth cell function and regulation of intestinal homeostasis in rats. Here, the absence of enteric food caused an up-regulation of lysozyme and rat α-defensins 5 and 8 (169). Remarkably, the authors noted an inverse correlation between lysozyme expression and Firmicutes abundance in the small intestine, implying a link between HDP function and the small intestinal microbiota (169). Regarding the contrasting results, the authors speculated that the main difference between both studies was that the rats fed with parenteral nutrition could still respond to changes in the microbiota, as the Paneth cells were still functional, as opposed to the altered granule physiology observed in starved mice (169). Indeed, Liang *et al*. expanded these observations in a longer mouse starvation study of 72 h and identified that the starved group showed a drop in *Reg3*β, *Reg3*γ and *Mmp7* gene expression after the first 48 h, and later an increased expression of *Mmp7* at 72 h (150). The authors investigated the V-shaped pattern of expression of *Mmp7* and were able to demonstrate that an initial drop in the microbial population caused the decreased expression 48 h post-starvation through Myd88-IL-22 signaling, and that the recovered expression 72 h post-starvation was regulated by the transcriptional repressor Hes1, controlled by the mTOR nutrient-sensing transcriptional regulator in response to the nutrient fluctuations (150). They additionally demonstrate that mTOR controlled Hes1 translation by sensing amino acids and glucose (150). Recently, intestinal neurons secreting vasoactive intestinal peptide (VIP) in response to food consumption were also implicated in the ILC3-mediated regulation of *Reg3*γ expression (170). Although the molecular food-sensing mechanism causing a VIPergic response remains unknown, this study evidences a link between neural-immune regulation of HDP expression and food intake.

Taken together, these studies suggest that microbiota as well as nutritional status play a role in HDP-modulation. Accordingly, these observations highlight the importance of the presence of food for the correct functioning of the antimicrobial program in the small intestine, as starvation has been associated with increased risk of bacterial translocation in patients receiving parenteral nutrition (171).

### The Effect of High Fat Diet (HFD) on HDP Expression

HFD is a general term for a diet with increased fat content. However, the amount and composition of fat content, as well as other dietary compounds can vary between studies, as can the length of the intervention. Such variations have the potential to change HDP expression pattern in the small intestine to varying degrees. Table 2 summarizes various mice and rat studies that investigated how different HFD treatments affect the expression of selected HDPs. Diets with 20–60% fat content substantially modify HDP expression patterns in the small intestine, whereas short term treatments (between 2 and 20 weeks) lowered HDP expression, which was in some cases also confirmed on protein levels. At the same time, longer treatments (>20 weeks) seemed to shift this pattern toward higher HDP expression levels.

**Table 2:** High fat diet (HFD)-modulation of host defense peptide expression in different studies.

**Duration of HFD treatment**	**Analyzed variables (% of fat content)**	**Changes in HDP expression compared with control group after HFD treatment**	**Intestinal area**	**Observations**	**References**
4 weeks	HFD (40%)	↓*Reg3γ* in all regions (except ileum), and ↓*Mmp7, Ang4, Lyz1, Defa3, Defa5, Defa20, Pla2g4a* in the ileum	Duodenum, jejunum and ileum	Increased bacterial colonization of the intervillous space and retention of Muc2 in the ileum after HFD treatment The HDP defects were corrected after administration of rosiglitazone, a Ppar-γ agonist, and after switching back to a standard diet	(172)
18–20 weeks	HFD (60%) and vitamin D deficiency	↓*Defa5, Defa1* and *Defb1*, and ↓*Mmp7* protein level	Ileum	Both the HFD and vitamin D deficiency induced insulin resistance and fatty liver	(173)
8 weeks + 19 days gestational period	Undernutrition (UN) HFD (60%) in the mother. Measured effects on the fetus's intestinal barrier function	Mothers UN ↓*Lyz2* and *Reg3γ* HFD ↓*Reg3γ, Muc2* and ↑*Lyz2* (also shown in a protein level) Fetus UN ↓*Muc2* HFD ↑*Lyz1* and *Reg3γ*	Mothers jejunum Fetal gut	Maternal UN was associated with reduced gut barrier function and integrity, fetal gut development and mucus production Maternal HFD was associated with increased barrier function and lysozyme production and with improved fetal gut barrier function and integrity	(174)
8 weeks	HFD (60%) Prebiotic treatment	↓*Reg3*γ, *Pla2g2, Lyz1* and *Ang4*	Jejunum	When compared to HFD, HFD-prebiotic treated mice had increased *Reg3γ* expression and epithelial cell turnover, decreased Firmicutes/Bacteroides ratio (evidencing an opposite effect in taxonomic shifts observed in gut microbiota), heightened SCFA production and reduced levels of plasma leptin	(175)
22–26 weeks	HFD (60%) Villin-Cre (VC) recombinase-mediated intestinal epithelial cell specific insulin receptor deletion (VC-IR knockout)	↓*Muc2* and ↑*Lyz1* and *Defa1a*. These last 2 HDP were not increased in VC-IR knockout	Jejunum	Deletion of the intestinal epithelial insulin receptor diminished the HFD-induced elevations in cholesterol and expression of Paneth cell peptides	(176)
8, 12, and 16 weeks	HFD (60%) different time points	↓*Lyz1, Reg3γ*, and *Ang4* at either 8, 12 and 16 weeks of HFD feeding	Small intestine (Ileum)	A HFD may stimulate intestinal inflammation via altering gut microbiota, which can occur prior to the increase of circulating inflammatory cytokines	(177)
20 weeks	HFD (60%) Supplementation with the polyphenol rutin or with rutin and polysaccharide inulin	↑*Defa5, Lyz1, Ang4*. No effect observed for Reg3γ. Lower protein levels of lysozyme	Small intestine (Ileum)	Rutin supplementation alleviated the increase of plasma triglycerides or leptin, attenuated the inflammatory response and improved ER stress caused by HFD. There was a positive correlation between increased expression of HDP with plasma LPS and inflammatory mediators, suggesting a link between Paneth cell HDPs and obesity-associated inflammation	(178)
2 weeks + 48 h induction with sodium taurocholate	HFD (20% saturated animal fat) and induction of acute necrotizing pancreatitis	↓*Lyz1* and *Defa5*	Ileum	Intestinal dysbiosis may contribute to the pathogenesis of intestinal barrier dysfunction in the context of acute necrotizing pancreatitis and hypertriglyceridemia	(179)^*^
14 weeks	HFD (60%) and dextran sodium sulfate (DSS) treatment	↓*Defa1, Defa4, CRS1C*, Lyz1 protein level	Ileum	The HFD treatment increased the susceptibility to DSS-induced colitis which was transferable through fecal transplantation and abolished after antibiotic treatment	(164)

*Unless specified, all studies were performed in mice*.

* *Study done in rats. Lyz1 (Paneth cell) and Lyz2 (myeloid specific)*.

HFDs, which also include the high fat/high carbohydrate WSDs, contain components that can directly (e.g., cholesterol or saturated fatty acids) or indirectly (e.g., TMAO) trigger inflammation (37). Specifically, saturated fatty acids present in WSDs, such as palmitic or stearic acid, induce ER stress, an insult that is sensed by macrophages (180). Also, a recent study demonstrated that HFD feeding caused a 50% increase in Lgr5+ intestinal stem cell numbers but at the same time a 23% reduction in Paneth cell numbers (181). Thus, we hypothesize that the initial reduction in HDP expression upon HFD feeding can be caused by the reduced numbers of Paneth cells (Table 2) (176, 179), and in addition by further detrimental effects, such as ER stress, which was previously linked to obesity and HDP malfunction (163). After prolonged treatment—for example, 5-month HFD intervention—a chronic inflammatory response is initiated in the gut that is reflected by the increased HDP expression.

Different pathways were suggested to control the dietary regulation of HDP expression. Tomas *et al*. focused on the pathological effects of a HFD in the small intestine, a region with key nutritional functions, and which seemed to have been understudied in the context of obesity, diabetes and metabolic syndrome (Table 2) (172). Among different detrimental effects, the authors identified that HFD treatment led to a downregulation of HDPs and the cystic fibrosis transmembrane conductance regulator (Cftr), as well as the peroxisome proliferator-activated receptor (Ppar)-γ, a nuclear receptor involved in lipid sensing and mucosal defense regulation (172, 182, 183). In addition, mice fed the HFD had increased numbers of bacteria in the intervillous zone in the small intestine—an otherwise sterile area. This aberrant colonization of bacteria in the villi was associated with the defective antimicrobial response in the ileum, and indeed, the administration of rosiglitazone, a Ppar-γ agonist, restored HDP expression levels; thus revealing the role of this receptor in controlling the antimicrobial function in the intestine (172).

While research on high-fat diets naturally focuses on the role of fat, these dietary compounds can also interact with other dietary factors. Su *et al*. demonstrated that both dietary fat and vitamin D can modulate *Defa* and *Mmp7* expression in mice. Vitamin D is a dietary component that supports mucosal barrier function when supplied in adequate amounts (184, 185). HFD treatment reduced the expression of *Defa5, Defa1, Defb1*, and the protein levels of Defa1 and Mmp7, and the reduction in HDP expression was even stronger in mice fed a diet low in vitamin D (Table 2) (173). Remarkably, the combination of a high-fat and vitamin D-deficient diet (called “the double hit model”) caused hepatic steatosis and insulin resistance. This effect was attributed to a state of dysbiosis in the small intestine, as a consequence of the low antimicrobial response. Accordingly, this hypothesis was confirmed by the administration of synthetic HD5 in the double hit model, which corrected the expression of HDPs, resolved the systemic inflammation and improved the observed insulin resistance (173).

Importantly, dietary supplements in the context of HFDs revealed additional factors with the potential of modulating mucosal barrier function. These factors included the polyphenol rutin and the prebiotics inulin and oligofructose (Table 2) (175, 178). While a 20 week HFD treatment elevated the expression of HDPs, both the administration of rutin or rutin and inulin reduced their transcription back to base expression levels (178). In addition, the administration of oligofructose significantly increased the expression level of *Reg3*γ (>50-fold), which was otherwise reduced by a HFD (175). These examples implicate polyphenols and prebiotics as keepers of intestinal homeostasis by correcting the aberrant expression of HDPs. As prebiotic interventions will promote the growth of beneficial bacteria, their HDP-modulatory effect might operate via modulation of microbial communities, which will be discussed in detail below.

Finally, a more indirect effect of HFD on HDP expression has been described recently. Upon consumption of a (high-fat) meal the host secretes bile acids to emulsify the fatty acids and facilitate their absorption. To test the effect of bile acids in mice, dietary supplementation with the primary bile-acid chenodeoxycholic acid (CDCA) was shown to induce the transcript levels of *Defa20, Reg3*β, and *Reg3*γ, and stimulate the production of Reg3β, and Reg3γ in different cell types along the villi in the ileum (186). While the effect on the microbiota was minimal and only increased relative abundance of Bacteroidetes, the increase in antimicrobial defenses protected the host from enteric *Salmonella* and *Citrobacter* infection, two microbes that are otherwise bile-resistant (186). Although the mechanism behind the induction could not be identified, this implicates bile acids as indirect effector molecules from the host that can regulate HDP expression and microbiota composition.

Altogether, food availability and specific dietary factors have the potential to significantly influence the intestinal antimicrobial defense system, an effect that is only starting to be understood. While most studies observed HDPs at the expression level, protein levels are expected to reveal more information about this function. Also, most of these studies were performed in mice and the effect of these factors in human trials remains to be demonstrated.

## Effect of Microbes and Their Metabolites on HDP Expression

Dietary factors elicit different responses that control microbial communities in the gut. Nevertheless, both the presence or absence of the microbiota as well as specific individual microbes have been linked to modulation of HDP expression. It is well-known that germ-free (GF) mice, which are completely devoid of a microbiota, undergo an incomplete development of the immune system (187, 188). As such, early studies of GF mice intestines revealed a decrease in expression of *Reg3*γ and *CRS4C*, as determined by total transcript copy number, when compared to conventional mice (23). Likewise, conventional mice had higher expression of *Reg3*γ, *Reg3*β, *RELM*β, and *CRS4C4* when compared to GF mice or to mice in which the microbiota had been depleted by an aggressive antibiotic regime (14). Similarly, observations in antibiotic depleted mice further corroborated the decreased expression of *Mmp7* and *Reg3*β in the ileum and of *Ang4, Pla2g2a, RELM*β, *Reg3*γ, and *Reg3*β in the colon (150, 189). Altogether, these observations seem to be a result of immature immune development in the absence of HDP-stimulating microbes.

### Microbes That Stimulate HDP Expression

Different studies have linked the enrichment of a particular microbe or the administration of a probiotic bacterium to increased expression of HDPs. For example, *B. thetaiotaomicron* has been shown to stimulate the production of Reg3γ and Ang4 in the small intestine and CRAMP in the colon (24, 127, 190). Moreover, *B. thetaiotaomicron* mediated the colonization resistance against *C. albicans* via induction of HIF-1α (190), a transcription factor involved in the activation of innate immune effectors that also regulates CRAMP in the murine gut and of HBD1 in humans (191, 192). Interestingly, this microbe has been described to be resistant to HDPs and could therefore survive the antimicrobial stimulation (16, 24). Similar to *B. thetaiotaomicron*, supplementation with *A. muciniphila* increased the expression of *Reg3*γ in the mouse colon when mice were fed a control diet, but not during treatment with a HFD, in which ileal *Pla2g2a, Defa*, and *Lyz1* were not stimulated by *A. muciniphila* (193). Also, monocolonization of mice with the probiotic *Bifidobacterium breve* NCC2950 induced *in vivo* and *in vitro* expression of *Reg3*γ, an effect that was mediated by the MyD88-Ticam axis of the TLR signaling pathway (194). Furthermore, Cazorla *et al*. isolated intestinal fluid from mice treated with the probiotic strains *L. casei* CRL431 and *L. paracasei* CNCM-I and showed that the extracted fluid had enhanced antimicrobial activity against *S. typhimurium* and *S. aureus* (195). Although this study did not measure HDP expression, the probiotic treatment increased the Paneth cell numbers in the crypts, hence suggesting an overall increase in the antimicrobial peptide function in the gut (195). In the same line, the probiotic strain *E. coli* Nissle 1917 evidenced a strong induction of HBD2 in *in vitro* studies (196), an effect that could be confirmed *in vivo* in a small human study, in which administration of an *E. coli* probiotic preparation (Symbioflor® 2) for 2–3 weeks increased the levels of this peptide in the feces (25). While the *in vitro* HBD2-inducing effect was caused by flagellin of *E. coli* Nissle 1917 (197), the *E.coli* strains in the probiotic preparation did not produce flagellin, thus suggesting that multiple mechanisms can mediate this effect and that they differ between individual strains (25).

### Microbial-Derived Components and Molecules Implicated in HDP Control

Specific microbial-derived molecules and metabolites have been associated with regulation of HDP function by stimulating their expression or by promoting their release. Early studies demonstrated that Paneth cells can respond directly to bacterial stimuli, either live or dead bacteria, lipopolysaccharide (LPS) or membrane components (21). This observation was performed in isolated crypt cells and shed light on defensin secretion capabilities. However, since a mucus layer covers the small intestinal epithelium, the probability of microbial derivates reaching the bottom of the crypts is expected to be low. Yet, the described Paneth cell response is likely a back-up response that could take place in the presence of severe mucosal damage or during abnormal microbial growth at this site. Still, isolated crypts may contain exposed basolateral receptors and are perhaps more responsive to bacterial stimuli. Accordingly, using mouse-derived small intestinal organoids, Farin *et al*. showed that Paneth cell degranulation did not occur after stimulation with bacterial agents, but rather after direct induction with IFN-γ or a supernatant derived from stimulated iNKT cull culture (138). Similarly, the SCFA butyrate was shown to directly enhance the production of Defa1 in isolated crypts from the small intestine (165), but it is unclear whether Paneth cells could be stimulated by this fermentation product in the small intestine *in vivo*, where the concentration of butyrate is relatively low (165, 198). Furthermore, by using a human-derived reporter cancer cell line, Sugi *et al*. investigated the transcription of *Defa5* after challenge with the bacterial ligands LPS, the synthetic lipopeptide P3CSK4 or with the bacterial metabolites acetate, lactate, butyrate, and propionate. Among these molecules, lactate strongly suppressed the transcription of *Defa5* while propionate and butyrate were suppressive only at a high concentration (9 mM) (198). However, this intestinal cell line represents absorptive epithelial cells, and their defensin expression capacities are lower when compared to Paneth cells. Nevertheless, lactate was found in high concentrations in the small intestine, suggesting it could suppress the transcription of *Defa5 in vivo* and thereby prevent the release of pyrogenic molecules and the probable aberrant activation of inflammation (198).

In addition to SCFA-mediated HDP regulation, microbial-derived tryptophan catabolites (e.g., indole or indole-3-aldehyde) can bind the aryl hydrocarbon receptor (AhR) and activate IL-22 secretion (199). The AhR is a transcriptional factor expressed by several immune cell types, including RORgt+ ILC3s, that is crucial for the control of intestinal homeostasis in a ligand dependent manner (200). Binding of microbial AhR ligands induced IL-22 secretion by ILCs and stimulated the expression of the HDPs *Lcn*-2, *S100A8*, and *S100A9*, which was shown to be protective against *C. albicans* infection (26).

As described in section 3, Levy *et al*. demonstrated the IL-18 mediated induction of colonic *ITLN1, RELM*β and angiogenins (27). By using colonic explants and colonic spheroids, the authors identified the microbiota-associated metabolite taurine, a bile acid conjugate, as the most potent activator of IL-18, which stimulated HDP expression via the activation of the NLRP6 inflammasome. Conversely, histamine and spermine (polyamine) featured the strongest suppression of IL-18. Furthermore, when taurine was administered to mice with dextran sodium sulfate (DSS)-induced colitis, this metabolite greatly improved colitis severity and weight loss, stressing its *in vivo* importance in the context of disease (27).

Consequently, the members of the microbiota play an essential role in the innate immune development and regulation of HDP function against pathogen infection. This effect may be mediated by the presence of beneficial microbes or by their associated metabolites. Identifying microbes or metabolites that have high potential to influence HDPs will aid in the selection of potential new probiotic strains. Furthermore, the use of such microbial metabolites could be exploited as a “postbiotic” treatment to control the interactions between host and microbiota (27).

## Diet-Microbiota-Host Defense Interaction

**Figure 4:**
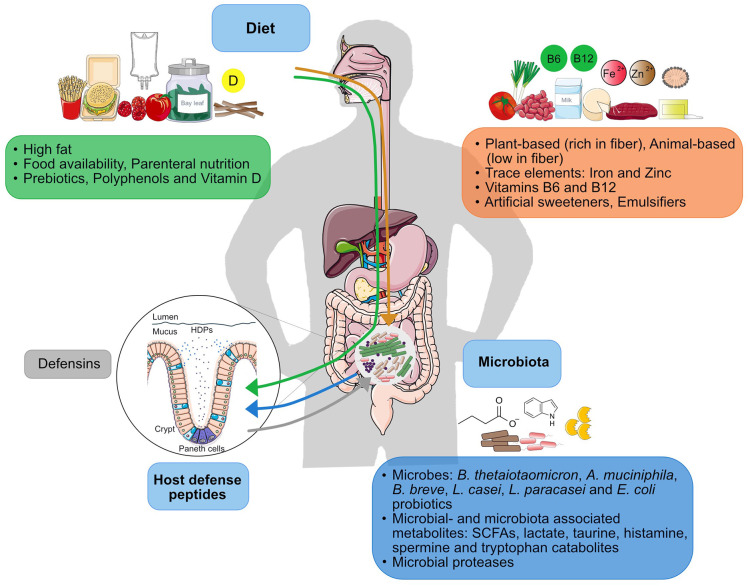
Factors within the diet-microbiota-HDP axis. The continuous interplay between microbiota and HDP (blue and gray arrows) is constantly influenced by diet. Effect of dietary factors on the microbiota is represented by the orange arrow and the effect of dietary factors on HDP function is represented by the green arrow.

Diet composition can define microbial communities in the intestine and the antimicrobial programming in the intestinal epithelium. Given the close interactions between microbes and HDPs, there is undoubtedly a close interrelationship of diet, HDPs, and the intestinal microbiota (Figure 4), in which it is difficult to define the extent to which each component is regulating each other.

The microbiota is a dynamic community and different types of diets change the microbiota composition. As we discussed, plant-based components seem to enrich beneficial microbes with the potential of improving immune function, whereas HFDs tend to enrich pro-inflammatory Proteobacteria communities. In both cases, however, HDP expression is also altered, but it is unclear if diet-dependent alterations of the microbiota composition can at least in part be caused by modulation of HDP expression. Most HFDs studies discussed here report that both the changes in HDP expression (Table 2) and microbiota alterations take place at the same time. However, by looking at different time-points, Guo *et al*. found that HFD-mediated alterations of the microbiota preceded changes in the levels of circulating inflammatory cytokines (Table 2) (177). Thus, HFD may presumably first change microbiota composition which in turn influences the immune response. In addition, diet composition and microbial metabolism will likely affect environmental conditions such as redox-potential and pH that can further mediate defensin function (18, 19). As a result of microbial fermentation, the pH in the gut becomes more acidic (from 6.5 to 5.5 as determined *in-vitro*), which can directly influence the activity of HDPs and favor the growth of butyrate-producing Firmicutes, such as *Roseburia spp*., while reducing the proliferation of the acid-sensitive *Bacteroides spp*. (201, 202).

Microbes can regulate HDP function directly through their structural components, through proteolytic activation/deactivation, and via their metabolites. However, defining the causative contribution of complex microbial communities on HDP expression is more challenging. Yet, a dysbiotic microbiota obtained from the caecum of SPF mice with ileitis was shown to transmit the inflammatory phenotype to genetically susceptible GF mice, which also led to a reduced expression of lysozyme, but not *Defa2* (203). The transferred dysbiotic microbiota was characterized by an increase in the relative abundance of *Clostridiales* and decreased abundance of *Porphyromonadacaeae* (order of Bacteroidales), suggesting a pro-inflammatory potential of these taxa (203). Remarkably, it was the caecal microbiota that could induce the defect in the small intestine, which is unexpected, as the small intestine possess its own characteristic microbial community.

When attempting to understand the role of microbes in controlling HDP function, the mucosa-associated microbiota is expected to have an even stronger effect on the host as the luminal microbiota, since it is in closer interaction with the epithelium. Furthermore, in the case of defensin function, the ileal microbiota is expected to have a stronger influence than the colonic community. Indeed, Su *et al*. observed in their HFD experiment a reduced HDP expression (Table 2) that was accompanied by changes in microbiota composition in the ileum (173). They observed a pronounced increase in Proteobacteria with members *Campylobacterales* and *Helicobacteraceae* (including the hepatotoxic *Helicobacter hepaticus*), a mild increase in Firmicutes and a reduction in Bacteroidetes. Thus, a closer examination of the missing microbial communities mentioned in these studies could help in identifying microbes with distinct HDP modulating function.

## Concluding Remarks

We are only beginning to understand how certain dietary components and microbes can signal to the epithelium and mediate HDP function. We discussed that the nutrient-sensing signaling mediators Ppar-γ, AhR, VDR, mTOR, and VIP-producing neurons are involved in diet-associated HDP control, while immune mediators in the TLR-Myd88/TRIF signaling pathway as well as the cytokines IFN-γ, IL-22, and IL-18 shape the microbe-dependent HDP regulation. However, more research is required to define the individual contribution of these different mediators to the complex regulation of intestinal HDP expression, and we believe that identifying strategies to fine-tune diet-HDP-microbiota interplay is a promising approach to strengthen the innate defenses in HDP-related disorders. In addition, further studies are required to understand whether HDPs can modulate and kill commensal bacteria in the gut or whether their activity is indeed limited to kill pathogenic microorganisms.

So, does an apple a day also keep the microbes away? Unfortunately, our current knowledge does not allow us to answer this question, but instead prompts us to ask other questions: Do we in fact want the microbes away from the intestinal mucosa, and thus eliminate beneficial microbes that stimulate HDPs? Or can we specifically fine-tune our defenses to target only pathogens and not commensals? Thus, rather than just relying on an apple, a diverse diet with different proportions of macronutrients and micronutrients is likely the food of choice to maintain a stable microbiota community that is separated from the host through a balanced HDP-microbiota homeostasis.

## Author Contributions

The manuscript was written and edited by FP-B and BS. FP-B generated the figures included in this study using templates from Servier Medical Art by Servier licensed under a Creative Commons Attribution 3.0 Unported License.

## Conflict of Interest

The authors declare that the research was conducted in the absence of any commercial or financial relationships that could be construed as a potential conflict of interest.
